# The uneven landscape of Swiss travel behavior: evidence of mobility inequality from the national microcensus

**DOI:** 10.1038/s44333-026-00085-5

**Published:** 2026-03-11

**Authors:** Matthias F. C. Hudecek, Julia K. Kammer, Corinne Moser

**Affiliations:** FHNW School of Applied Psychology, University of Applied Sciences and Arts Northwestern Switzerland, Olten, Switzerland

**Keywords:** Environmental social sciences, Geography, Geography

## Abstract

This study shows that mobility in Switzerland is highly unequal. Based on Swiss Mobility and Transport Microcensus data (years 2015 and 2021), we find that a small percentage of individuals accounts for a disproportionately large share of total travel. Machine learning reveals that sociodemographic and spatial features have limited predictive power for daily domestic travel distance. The findings highlight the need for more nuanced perspectives that go beyond population averages.

## Introduction

Climate change is a pressing global challenge requiring drastic reductions in greenhouse gas (GHG) emissions^1^. Switzerland emits approximately 13 tons of CO₂ per capita each year—more than twice the global average of 6 tons^2^. Mobility is a major driver of these emissions. The transport sector accounts for nearly 33% of total CO₂ emissions, with passenger transport alone responsible for over 75% of Switzerland’s transport-related emissions^3^. While other sectors (e.g., industry and buildings) have significantly cut emissions, transport-related emissions have remained almost at the same level since 1990^4^. Therefore, significant changes in individual mobility behavior, supported by targeted policy measures, are essential^5^. Following the polluter-pays principle^6^, one approach is to target individuals who disproportionately contribute to GHG emissions^7^. Research indicates that mobility-related emissions are heavily unequally distributed. A small fraction of individuals—typically the top 10–20%—are responsible for an inordinately large share of transport emissions^8–10^, thus resembling the concept of global carbon inequality^11^. These individuals are more likely to be male^12,13^, more affluent^9,10^, and to live in suburban areas^9,13^. However, other studies suggest the need for a more nuanced perspective regarding spatial characteristics, as individuals in large urban areas were also found to emit higher levels of GHG emissions compared to the average^14^.

The present study investigates whether similar patterns of mobility-related inequality are present in Switzerland. The goal is to establish the existence and extent of domestic mobility inequality within Switzerland. Leveraging data of the Swiss Mobility and Transport Microcensus (MTMC) from 2015 (*N* = 57,090) and 2021 (*N* = 55,018), we analyze the distribution of daily domestic travel distance across individuals to determine whether a small group of high travelers accounts for a significant share of total mobility. In addition, we test the predictive power of spatial (e.g., level of urbanization) and sociodemographic characteristics (e.g., gender, income) for variations in daily domestic travel distances.

**Fig. 1:**
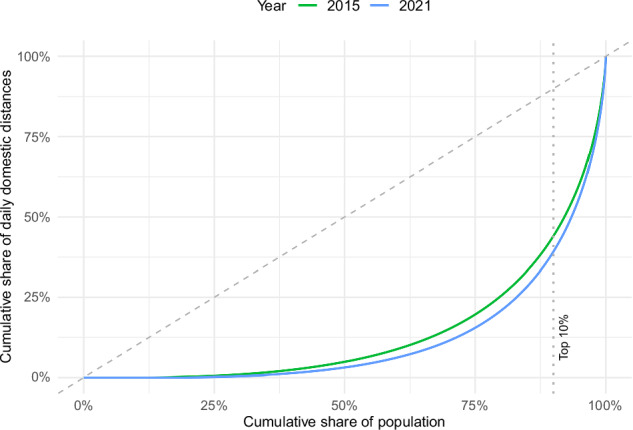
Lorenz curves of daily domestic travel distances in Switzerland for the years 2015 and 2021. The dotted vertical line marks the 90th percentile of the population.

The distribution of daily domestic travel distances in Switzerland demonstrates significant inequality among the population (see Fig. 1). In both years, a small percentage of individuals accounted for a disproportionately large share of the total mobility. In 2015, the upper 10% contributed 53% of total daily domestic distance, followed by 56% in 2021 (see Table 1; full descriptive statistics are provided in Supplementary Materials A). Thus, the top 10% of the population engaged in mobility contribute more to the daily domestic travel distance as the other 90% combined—not counting those who reported no mobility. The Gini coefficients highlight this pattern (2015: 0.72; 2021: 0.76). Non-overlapping 95% bootstrap confidence intervals for each year’s Gini coefficient (2015: CI = [0.7145–0.7211]; 2021: CI = [0.7560–0.7623]) show that the slight increase in inequality over the years is statistically significant.

**Table 1:** Descriptive statistics of daily domestic travel distances (in km) among the groups for the years 2015 and 2021

Group	2015			2021		
	*M*	SD	*n*	*M*	SD	*n*
0	0	0	6649	0	0	9581
1	0.97	0.48	5045	0.70	0.32	4544
2	2.69	0.55	5044	1.86	0.37	4544
3	4.94	0.76	5044	3.41	0.53	4544
4	8.03	1.03	5044	5.65	0.78	4544
5	12.29	1.46	5044	9.02	1.16	4544
6	18.53	2.17	5044	14.01	1.81	4544
7	28.28	3.57	5044	22.40	3.10	4544
8	44.57	6.19	5044	36.93	5.59	4543
9	76.50	13.63	5044	66.46	12.68	4543
10	220.07	156.23	5044	203.02	147.55	4543

Values refer to individual-level travel within Switzerland based on the weighted MTMC data. Group 0 = no travel; groups 1–10 = deciles of the traveling population. A complete overview of all descriptive statistics can be found in Supplementary Materials A.

An analysis by mode of transport reveals that motorized individual vehicles (MIV) account for the majority of distance traveled (see Table 2) and mainly drive the overall inequalities. An exponential increase in MIV distance is observed across groups; similarly, public transport (PT) distances grow exponentially toward the top deciles. In contrast, active transport (AT) is distributed evenly across all groups (see full descriptive statistics in Supplementary Materials B).

**Table 2:** Average modal share in % of total daily travel distance across mobility groups (Groups 1–10) for the years 2015 and 2021

Group	2015			2021		
	MIV	PT	AT	MIV	PT	AT
1	19.59	1.03	77.32	11.43	1.43	85.71
2	32.34	4.83	60.97	22.58	3.23	71.51
3	41.09	10.32	47.17	33.14	6.16	58.94
4	50.56	12.70	35.87	44.07	9.91	44.42
5	57.77	15.54	25.71	53.22	11.64	34.15
6	64.27	16.24	18.56	61.96	12.71	24.34
7	68.10	17.64	13.40	68.17	13.39	17.41
8	70.43	19.65	9.00	71.68	16.14	11.45
9	69.42	23.70	5.88	72.84	19.55	6.74
10	66.22	28.75	2.37	72.96	22.78	2.70

Values represent the proportional contributions (in %) of each individual’s total domestic travel distance attributed to motorized individual transport (MIV), public transport (PT), and active transport (AT) based on the weighted MTMC data. The values for MIV, PT, and AT do not sum to 100% because trips categorized as ‘other modes’ (e.g., boats or other vehicle-like devices) are reported separately in Supplementary Materials B. Non-mobile individuals (Group 0) are excluded. Shares are based on the relative contribution of each mode to the total distance traveled per person.

We further analyzed the identified groups with respect to spatial characteristics. Urban residents are more prevalent in lower mobility groups and gradually decrease as the mobility increases. Conversely, rural municipalities display an opposite trend. Their share remains steady among low mobility groups and then rises sharply toward the top deciles. This pattern is consistent across both years. Still, the results do not suggest that the differences in mobility behavior among the groups can be entirely explained by a simple urban–rural divide. Although the proportion of urban residents decreases towards the top deciles, it still accounts for 43% in the top decile. A further graphical analysis plotting daily domestic travel distances on a map of Switzerland at the municipal level supports this assessment (see Supplementary Materials C).

Regarding sociodemographic characteristics, analyses reveal consistent differences. Women are overrepresented among individuals with low daily travel distances, while men are more common in the highest mobility deciles. The average age is highest among individuals with no reported mobility, and there is a trend of decreasing age in higher mobility deciles. Higher mobility is positively associated with socioeconomic status: the most mobile individuals are more likely to report monthly household incomes above CHF 12,000 and to hold tertiary qualifications. Conversely, low-income and less-educated individuals tend to be concentrated in the low mobility segments (see full descriptive statistics in Supplementary Materials D).

**Fig. 2:**
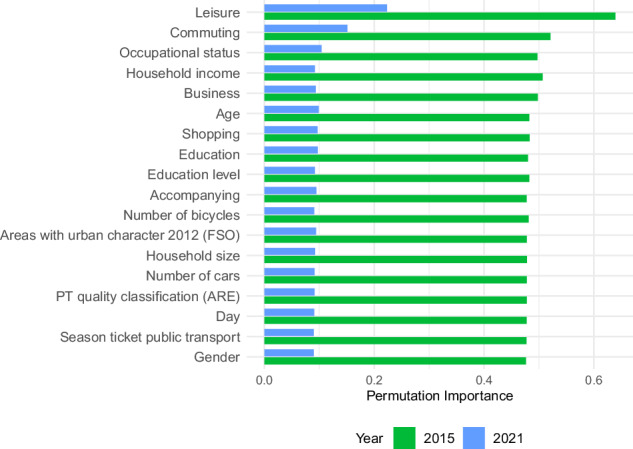
Relative permutation importance for the years 2015 and 2021.

**Fig. 3:**
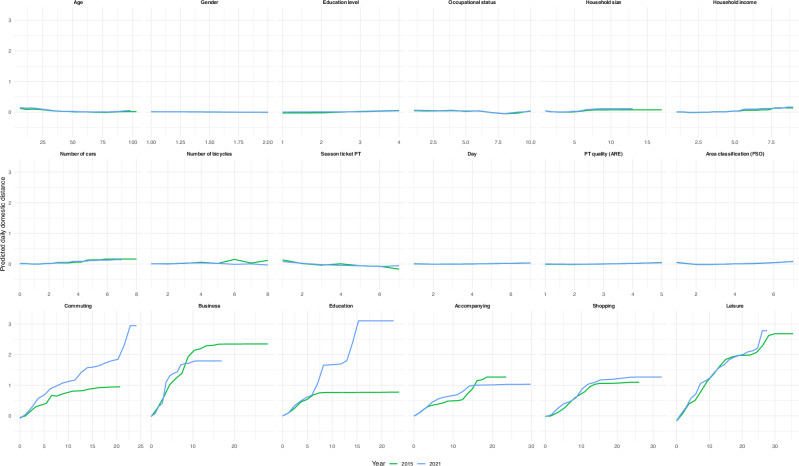
Partial dependence plots for the years 2015 and 2021. Partial dependence plots display the marginal effect of each predictor on the standardized daily travel distance. *X*-axis shows the raw predictor scales or, for factors, their integer codes. *Y*-axis shows the model’s average predicted z-distance, with all other covariates held at their observed values. Calculation of daily domestic travel distances based on the weighted MTMC data.

The *R*^2^ values of the random forest regression models predicting daily domestic travel distances are weak to moderate (2015: *R*^2^ = 0.217; 2021: *R*^2^ = 0.223). Top predictors based on relative permutation importance (see Fig. 2) across both datasets include leisure activities and commuting as reasons for mobility, along with household income (2015) and occupational status (2021) as sociodemographic characteristics. Spatial characteristics (quality of PT infrastructure and degree of urbanization) are among the predictors with lower relative permutation importance across both datasets. Partial dependence plots further visualize the relationship between each predictor and daily domestic distance (see Fig. 3). However, it is worth noting that the differences in permutation importance of the predictors within each dataset are relatively small. A full overview of the descriptive statistics for the predictor variables is provided in Supplementary Materials D.

Taken together, these results provide three key findings. First, mobility behavior is unevenly distributed across Switzerland. This aligns with research showing that GHG emissions in general^15^ and mobility behavior specifically (e.g., Canada^10^, France^13^, Germany^9^, UK^8^) are marked by significant inequality. Overall, the level of mobility-related inequality in Switzerland is slightly higher but still comparable to that in Germany, where the top 10% of emitters were found to account for 51% of total emissions^9^. Notably, the inequality in Switzerland is primarily driven by MIV and long-distance train travel, while AT is relatively evenly distributed among all groups.

National mobility surveys in Switzerland, as in many countries, are crucial for shaping transport and climate policy by providing comprehensive insights into individual travel behavior^16^. To date, official reports at the national^17,18^ and cantonal level^19,20^ have primarily focused on average metrics such as mean travel distances, modal shares, and differences between groups (e.g., gender or urban-rural), while giving limited attention to the inequality of mobility. Media coverage has followed suit, emphasizing national trends but rarely exploring disparities within the population^21,22^. The results of our study emphasize that a more differentiated perspective on who contributes to what level of mobility may be beneficial, especially when developing measures to reduce transport-related GHG emissions more effectively. Second, this mobility-related inequality in Switzerland has increased over time. Although mobility declined in 2020 (see Supplementary Materials on OSF) due to the COVID-19 pandemic, inequality increased in 2021 compared to 2015. Interestingly, all groups showed a reduction in traveled distances. However, the decrease was more significant in the lower deciles compared to the top deciles. For example, daily domestic travel distance in the top decile dropped by 8% from 2015 to 2021, while the average decline for the other deciles was 25%. This suggests that those in the top decile may be more resistant to changing their routines (e.g., commuting less due to remote work) while having the resources to cope with restrictions and maintain their established mobility behavior. Third, mobility-related, sociodemographic, and spatial characteristics are associated with mobility behavior, reflecting trends similar to existing literature^9,10,12,13^. However, taking the machine learning approach revealed that these variables have only weak predictive power for the daily domestic travel distance. Thus, our findings caution against overly simplistic classifications like ‘the typical high-mobility individual.’ A promising direction for future research is to investigate the motivations and behavioral mechanisms of highly mobile individuals. Classification models could be used to identify which characteristics best predict exceptionally high mobility, and qualitative approaches (e.g., interviews) may provide additional insight into the underlying decision-making processes and mobility needs. From a practical perspective, policy measures and interventions are necessary to engage those most responsible for mobility-related emissions. Instead of targeting specific individuals based on sociodemographic or spatial characteristics, the measures should be designed to naturally and progressively target high-mobility behaviors while minimizing impact on those who engage in lower mobility. At the same time, people who engage in low mobility behaviors might be rewarded or incentivized not to switch to higher-distance travel.

Some limitations of the study should be noted. First, the applied machine learning approach does not allow for causal inference. While random forests help to understand relationships between predictors and mobility behavior, they cannot establish causal effects. Future research should address this, for example, by using longitudinal data. Second, our study focused on domestic travel distances since the MTMC primarily provides detailed, reliable data on trips within Switzerland. Only a third of MTMC participants answered questions about international air travel in 2015 and 2021. Exploratory analyses of air travel distances show a similar, although less pronounced, pattern of inequality. Again, the top deciles contribute significantly more to the total air travel distances. A full overview is provided in Supplementary Materials E.

In conclusion, our study shows the unequal distribution of mobility behavior in Switzerland using data from the Swiss MTMC. A small group of individuals accounts for a disproportionate share of total mobility. This highlights the need for a more nuanced view of who is responsible for what amounts of mobility. While aggregated metrics like average travel distances help describe the overall situation, our data suggest that high-mobility individuals in the top deciles need targeted efforts to reduce their GHG emissions. In addition, random forests show that sociodemographic, spatial, and mobility-related characteristics only have weak predictive power in explaining daily domestic distance.

## Methods

### Data

We used the data of the Swiss Mobility and Transport Microcensus (MTMC) from the years 2015 (*N* = 57,090) and 2021 (*N* = 55,018) to analyze the distribution of daily domestic travel distance across individuals to determine whether a small group of high travelers accounts for a significant share of total mobility. The Swiss Mobility and Transport Microcensus (MTMC) is carried out by the Swiss Federal Statistical Office (FSO) in collaboration with the Swiss Federal Office for Spatial Development (ARE). It is the most important and comprehensive survey on mobility behavior of the Swiss population. The first survey was conducted in 1974, and it has been repeated every five years since then. It captures detailed information on individual travel patterns, transport modes, and socio-demographic factors. The data can be requested from FSO and used under license for further analysis. In our study, we examined the datasets from 2015 and 2021. Data collection in 2020 was stopped before completion and rescheduled for 2021 due to the COVID-19 pandemic. In 2021, the data collection was resumed and fully completed. During that period, there were no mobility restrictions in Switzerland. However, the potential impact of the COVID-19 pandemic on the mobility behavior should still be considered.

The data collection of the MTMC follows a complex procedure. Participants are randomly selected and interviewed about their mobility on a randomly selected day using the CATI method (computer-assisted telephone interviewing). Every outdoor distance of 25 m or more had to be reported. In addition, participants are asked a variety of questions covering mobility behavior, sociodemographics, and specific attitudes (e.g., car sharing, public transportation). Participation is voluntary. The complete list of items is available on the website of the FSO (2015: https://www.bfs.admin.ch/bfs/de/home/statistiken/mobilitaet-verkehr/erhebungen/mzmv.assetdetail.5606052.html; 2021: https://www.bfs.admin.ch/bfs/de/home/statistiken/mobilitaet-verkehr/erhebungen/mzmv.assetdetail.24845295.html). Households and individuals are weighted using a well-established method (CALMAR2 method in SAS) to ensure that the results are representative of the entire Swiss population. According to the official reports of the FSO, the data from the 2015 and 2021 MTCM are representative of the Swiss population^17,18^. Only fully completed interviews were included in the final datasets. Person and household weights are part of the datasets provided by the FSO. We used the *weighted* data for the main analyses in this paper. Although this method ensures representativeness for the Swiss population, it also results in unrealistically high travel distances for some individuals in the highest decile. Therefore, we also provide the results for the unweighted data in the online supplementary materials on OSF (https://osf.io/mk8st). The full report on the MTMC methodology, including the weighting process and calibrating variables, can be found on the website of the FSO (2015: https://www.bfs.admin.ch/bfs/de/home/statistiken/mobilitaet-verkehr/erhebungen/mzmv.assetdetail.4262242.html; 2021: https://www.bfs.admin.ch/bfs/de/home/statistiken/mobilitaet-verkehr/erhebungen/mzmv.assetdetail.24266729.html).

### Analyses procedure

The analyses involved three steps and combined descriptive statistics and inequality metrics, such as Gini coefficients and Lorenz curves, with a predictive modeling approach using Random Forest regression. All analyses were carried out in R (R version 4.4.2; R Studio version 2024.12.0 + 467).

First, respondents were divided into deciles according to their daily domestic travel distance using the ntile function of the dplyr package. All individuals who did not engage in any mobility were classified as an additional zero-mobility group. Gini coefficients with bootstrap confidence intervals (5000 draws) were used to assess inequality for each year (ineq package). Additionally, Lorenz curves were plotted to illustrate inequality visually. The groups resulting from the discretization were then described based on the transport mode used, along with their spatial and sociodemographic characteristics.

Second, to visualize regional mobility differences, we created a map of Switzerland for each year using ThemaKart 2025 from the FSO with the bfsMaps package in R. For each year, the mean daily domestic travel distance was calculated at the municipality level based on the official 2025 FSO classification. To comply with MTMC data use regulations of the FSO and to ensure anonymity, only municipalities with a minimum sample size of *n* ≥ 10 were included in the analysis. Municipalities with fewer than 10 observations were excluded from the illustration and shown in a white-dotted style, while municipalities with no available data were displayed with cross-hatching.

Third, we trained random forest regression models (ranger function and package) to test sociodemographic and spatial characteristics, as well as reasons for mobility, as predictors for daily domestic travel distance. Random Forests are a non-parametric machine learning method capable of modeling non-linear and interaction effects without assuming a specific functional form^23^. Given the non-normal distribution of the data, the potential non-linear relationships between certain predictor variables and the outcome (e.g., age^24^), along with the different scales and types of variables, this approach is especially well-suited. Random Forests are widely used in transportation^25,26^ and social-behavioral research^27,28^ to investigate variable importance in complex datasets. Following the procedure of Pavlović et al.^29^., partial dependence plots and permutation importance metrics were calculated. To optimize the random forest models, we tested 1000 and 2000 trees, used *R*^2^ as the accuracy metric, and extracted permutation-based variable importance. The number of variables considered at each split ranged from 6 to 18 (with an increment of one), and splits were based on variance. Minimum node sizes varied from 3 to 99 (with an increment of three). To ensure robustness, a holdout sample (20%) served as the test set for evaluating *R*^2^. It is important to note that absolute values of permutation importance are not directly comparable across datasets. We thus focused our interpretation on relative patterns across years (e.g., which predictors consistently rank high or low). The following 18 variables were used as predictors:

#### Sociodemographic variables

Age was assessed as a numeric variable ranging from 6 to 103 years. *Gender* was recorded as a binary variable with two levels: female and male. *Education* was measured using four categories: below 15 years, primary education, secondary education, and tertiary education. *Occupational status* was assessed with ten categories: self-employed; family member working in the family business; employee; apprentice; unemployed; non-working person in education or training; non-working person in retirement; disabled non-working person; non-working housewife or non-working househusband; and other non-working person (for respondents aged 15 years or older). *Household size* was captured as a numeric variable ranging from 1 to 17 persons. *Household income* was assessed using nine categories: <2000; 2000–4000; 4001–6000; 6001–8000; 8001–10,000; 10,001–12,000; 12,001–14,000; 14,001–16,000; and >16,000 CHF. The *number of cars* in the household was measured as a numeric variable ranging from 0 to 8. *The number of bicycles* was also assessed as a numeric variable, ranging from 0 to 7. Car and bike ownership were capped at the 99.9th percentile to exclude implausibly high numbers (e.g., a maximum of 51 cars in 2015). *Day of the week* was included as a factor with seven levels representing Monday through Sunday. *Season ticket ownership* was assessed as a categorical variable with seven levels. The levels included the following types of public transport season tickets: GA Travelcard, Half-Fare Travelcard/Half-Fare Youth Travelcard, Regional Network Pass (i.e., zone-based pass), Point-to-point Travelcard, seven25 Travelcard/Gleis 7 Card, Junior Travelcard/Children’s Co-Travelcard, and Other Season Ticket (e.g., Modular Travelcard).

#### Spatial characteristics

The ARE public transport (PT) classification was used to assess *PT quality*. This classification considers the type of available PT, PT frequency, and the distance to the nearest PT stop, resulting in five quality levels: Class A (very good accessibility), Class B (good access), Class C (average access), Class D (poor access), and “No quality class” (marginal or no public transport access). The FSO classification of “Areas with urban character 2012” was applied to examine the *degree of urbanization*. This classification is based on a complex procedure and distinguishes seven levels: core municipality of an agglomeration (core city), core municipality of an agglomeration (main core), core municipality of an agglomeration (secondary core), municipality in the agglomeration belt, multi-oriented municipality, core municipality outside agglomerations, and rural municipality without urban character.

#### Reasons for mobility

The daily number of trips was used to assess a person’s mobility purposes. The MTMC differentiates six categories: *commuting* (numeric, range 0–12), *education* (numeric, range 0–8), *shopping* (numeric, range 0–11), *business* (numeric, range 0–8), *leisure* (numeric, range 0–14), and *accompanying* (numeric, range 0–13).

## Supplementary information

Supplementary Materials

## Data Availability

The data that support the findings of this study are available from the Swiss Federal Statistical Office (FSO). However, restrictions apply to the availability of these data, which were used under license for the current study, and so are not publicly available. The FSO determines cases for the exception to data disclosure. Alternatively, the data may be requested directly from the FSO for scientific purposes.

## Code availability

The R code used to analyze the data of this study is publicly available on the Open Science Framework at https://osf.io/mk8st.
