# Supplementary material for: The uneven landscape of Swiss travel behavior: evidence of mobility inequality from the national microcensus
Source: NPJ Sustain Mobil Transp. 2026 Mar 11;3(1):18. doi: 10.1038/s44333-026-00085-5 (PMC12979185; doi:10.1038/s44333-026-00085-5)

Supplementary Materials A

The Uneven Landscape of Swiss Travel Behavior: Evidence of Mobility Inequality from the National Microcensus

Table S1

Descriptive statistics of daily domestic travel distances (in km) among the groups.

Group	Min	Q1	Q2	Q3	Max	M	SD	n	Share
**2015**									
0	0	0	0	0	0	0	0	6,649	0
1	0.01	0.58	0.98	1.38	1.79	0.97	0.48	5,045	0.23
2	1.79	2.21	2.67	3.17	3.69	2.69	0.55	5,044	0.65
3	3.69	4.27	4.91	5.59	6.35	4.94	0.76	5,044	1.19
4	6.35	7.13	8.00	8.91	9.91	8.03	1.03	5,044	1.92
5	9.91	11.01	12.20	13.54	15.02	12.29	1.46	5,044	2.95
6	15.02	16.60	18.43	20.36	22.58	18.53	2.17	5,044	4.45
7	22.58	25.10	28.08	31.23	34.93	28.28	3.57	5,044	6.78
8	34.93	39.12	44.05	49.78	56.33	44.57	6.19	5,044	10.69
9	56.34	64.69	74.39	87.41	104.60	76.50	13.63	5,044	18.35
10	104.60	129.07	167.96	253.09	2,664.10	220.07	156.23	5,044	52.79
**2021**									
0	0	0	0	0	0	0	0	9,581	0
1	0.02	0.45	0.72	0.97	1.24	0.70	0.32	4,544	0.19
2	1.24	1.53	1.84	2.18	2.54	1.86	0.37	4,544	0.51
3	2.54	2.94	3.39	3.85	4.39	3.41	0.53	4,544	0.94
4	4.39	4.97	5.62	6.30	7.10	5.65	0.78	4,544	1.55
5	7.10	8.00	8.97	10.00	11.15	9.02	1.16	4,544	2.48
6	11.15	12.42	13.86	15.48	17.46	14.01	1.81	4,544	3.85
7	17.46	19.72	22.19	25.03	28.26	22.40	3.10	4,544	6.16
8	28.27	31.98	36.32	41.51	47.86	36.93	5.59	4,543	10.16
9	47.86	55.33	64.79	76.79	92.57	66.46	12.68	4,543	18.29
10	92.57	115.57	154.04	229.87	2,288.39	203.02	147.55	4,543	55.86

Note. Values refer to individual-level travel within Switzerland based on the weighted MTMC data. Group 0 = no travel; groups 1–10 = deciles among the traveling population. Share = share of total mobility per year in %.

Supplementary Materials B

The Uneven Landscape of Swiss Travel Behavior: Evidence of Mobility Inequality from the National Microcensus

Table S2

Descriptive statistics of daily domestic travel distances (in km) among the groups for motorized individual vehicles (MIV).

Group	Min	Q1	Q2	Q3	Max	M	SD	n	Share
**2015**									
0	0	0	0	0	0	0	0	6,649	0
1	0	0	0	0	1.79	0.19	0.43	5,045	19.59
2	0	0	0	1.96	3.69	0.87	1.21	5,044	32.34
3	0	0	0	4.28	6.35	2.03	2.27	5,044	41.09
4	0	0	4.57	7.47	9.91	4.06	3.68	5,044	50.56
5	0	0	9.34	11.90	15.02	7.10	5.51	5,044	57.77
6	0	0.09	15.39	18.29	22.58	11.91	8.00	5,044	64.27
7	0	6.63	23.95	28.37	34.93	19.26	11.95	5,044	68.10
8	0	13.76	38.12	45.42	56.33	31.39	18.93	5,044	70.43
9	0	15.64	63.05	78.45	104.53	53.11	33.77	5,044	69.42
10	0	12.07	126.74	191.48	1,821.81	145.74	149.22	5,044	66.22
**2021**									
0	0	0	0	0	0	0	0	9,581	0
1	0	0	0	0	1.23	0.08	0.25	4,544	11.43
2	0	0	0	0.62	2.54	0.42	0.75	4,544	22.58
3	0	0	0	2.70	4.39	1.13	1.52	4,544	33.14
4	0	0	1.53	5.08	7.10	2.49	2.62	4,544	44.07
5	0	0	5.97	8.63	11.14	4.80	4.19	4,544	53.22
6	0	0	11.39	13.80	17.46	8.68	6.27	4,544	61.96
7	0	4.36	18.82	22.69	28.26	15.27	9.67	4,544	68.17
8	0	15.99	31.54	37.96	47.86	26.47	15.68	4,543	71.68
9	0	27.43	55.58	69.80	92.57	48.41	29.22	4,543	72.84
10	0	65.58	120.51	186.99	2,185.36	148.12	149.27	4,543	72.96

Note. Values refer to individual-level travel within Switzerland based on the weighted MTMC data. Group 0 = no travel; groups 1–10 = deciles among the traveling population.

Table S3

Descriptive statistics of daily domestic travel distances (in km) among the groups for public transport (PT).

Group	Min	Q1	Q2	Q3	Max	M	SD	n	Share
				**2015**					
0	0	0	0	0	0	0	0	6,649	0
1	0	0	0	0.00	1.76	0.01	0.10	5,045	1.03
2	0	0	0	0.00	3.67	0.13	0.49	5,044	4.83
3	0	0	0	0.00	6.31	0.51	1.25	5,044	10.32
4	0	0	0	0.00	9.51	1.02	2.21	5,044	12.70
5	0	0	0	0.00	14.46	1.91	3.74	5,044	15.54
6	0	0	0	0.00	22.31	3.01	5.78	5,044	16.24
7	0	0	0	0.69	33.86	4.99	9.56	5,044	17.64
8	0	0	0	8.79	55.49	8.76	15.88	5,044	19.65
9	0	0	0	38.84	103.09	18.13	30.18	5,044	23.70
10	0	0	0	106.54	2,588.52	63.26	134.41	5,044	28.75
				**2021**					
0	0	0	0	0	0	0	0	9,581	0
1	0	0	0	0	1.09	0.01	0.06	4,544	1.43
2	0	0	0	0	2.24	0.06	0.28	4,544	3.23
3	0	0	0	0	4.14	0.21	0.67	4,544	6.16
4	0	0	0	0	7.06	0.56	1.42	4,544	9.91
5	0	0	0	0	10.64	1.05	2.42	4,544	11.64
6	0	0	0	0	17.11	1.78	3.96	4,544	12.71
7	0	0	0	0	27.73	3.00	6.78	4,544	13.39
8	0	0	0	0	47.00	5.96	12.39	4,543	16.14
9	0	0	0	2.96	91.11	12.99	24.58	4,543	19.55
10	0	0	0	41.09	2,233.50	46.25	106.11	4,543	22.78

Note. Values refer to individual-level travel within Switzerland based on the weighted MTMC data. Group 0 = no travel; groups 1–10 = deciles among the traveling population.

Table S4
Descriptive statistics of daily domestic travel distances (in km) among the groups for active transport (AT).

Group	Min	Q1	Q2	Q3	Max	M	SD	n	Share
					**2015**				
0	0	0	0	0	0	0	0	6,649	0
1	0	0.28	0.73	1.20	1.79	0.75	0.54	5,045	77.32
2	0	0.29	1.93	2.64	3.69	1.64	1.21	5,044	60.97
3	0	0.14	1.77	4.29	6.35	2.33	2.11	5,044	47.17
4	0	0	1.54	5.76	9.90	2.88	3.16	5,044	35.87
5	0	0	1.27	4.47	15.00	3.16	4.14	5,044	25.71
6	0	0	1.09	4.09	22.55	3.44	5.32	5,044	18.56
7	0	0	1.00	3.97	34.77	3.79	6.83	5,044	13.40
8	0	0	0.94	3.83	56.32	4.01	8.39	5,044	9.00
9	0	0	1.03	4.22	103.82	4.50	10.72	5,044	5.88
10	0	0	1.02	4.91	436.46	5.22	15.27	5,044	2.37
					**2021**				
0	0	0	0	0	0	0	0	9,581	0
1	0	0.32	0.61	0.91	1.24	0.60	0.37	4,544	85.71
2	0	0.61	1.51	1.97	2.54	1.33	0.81	4,544	71.51
3	0	0.24	2.58	3.33	4.39	2.01	1.52	4,544	58.94
4	0	0	1.63	4.92	7.10	2.51	2.44	4,544	44.42
5	0	0	1.38	6.45	11.14	3.08	3.60	4,544	34.15
6	0	0	1.04	4.48	17.44	3.41	4.88	4,544	24.34
7	0	0	0.77	4.18	28.08	3.90	6.66	4,544	17.41
8	0	0	0.67	3.58	47.85	4.23	8.72	4,543	11.45
9	0	0	0.64	3.76	90.54	4.48	11.09	4,543	6.74
10	0	0	0.37	4.08	375.40	5.48	19.05	4,543	2.70

Note. Values refer to individual-level travel within Switzerland based on the weighted MTMC data. Group 0 = no travel; groups 1–10 = deciles among the traveling population.

Table S5

Descriptive statistics of daily domestic travel distances (in km) among the groups for other modes (e.g., boats or other vehicle-like devices).

Group	Min	Q1	Q2	Q3	Max	M	SD	n	Share
					**2015**				
0	0	0	0	0	0	0	0	6,649	0
1	0	0	0	0	1.75	0.02	0.15	5,045	0.02
2	0	0	0	0	3.67	0.05	0.33	5,044	0.02
3	0	0	0	0	6.11	0.07	0.47	5,044	0.01
4	0	0	0	0	9.79	0.07	0.62	5,044	0.01
5	0	0	0	0	14.45	0.12	0.99	5,044	0.01
6	0	0	0	0	21.80	0.17	1.41	5,044	0.01
7	0	0	0	0	33.71	0.25	2.25	5,044	0.01
8	0	0	0	0	55.61	0.41	3.60	5,044	0.01
9	0	0	0	0	98.96	0.76	6.17	5,044	0.01
10	0	0	0	0	1,214.65	5.85	48.78	5,044	0.03
					**2021**				
0	0	0	0	0	0.00	0.00	0.00	9,581	0
1	0	0	0	0	1.22	0.01	0.11	4,544	0.01
2	0	0	0	0	2.52	0.04	0.26	4,544	0.01
3	0	0	0	0	4.38	0.06	0.42	4,544	0.01
4	0	0	0	0	7.10	0.09	0.62	4,544	0.01
5	0	0	0	0	10.85	0.10	0.78	4,544	0.01
6	0	0	0	0	16.44	0.14	1.12	4,544	0.01
7	0	0	0	0	27.11	0.22	1.83	4,544	0.01
8	0	0	0	0	47.76	0.27	2.74	4,543	0.01
9	0	0	0	0	84.30	0.58	5.14	4,543	0.01
10	0	0	0	0	1,102.02	3.18	33.72	4,543	0.02

Note. Values refer to individual-level travel within Switzerland based on the weighted MTMC data. Group 0 = no travel; groups 1–10 = deciles among the traveling population.

Supplementary Materials C

The Uneven Landscape of Swiss Travel Behavior: Evidence of Mobility Inequality from the National Microcensus

Figure S1

Relative distribution of urban types across mobility groups for the year 2015. Urban types are based on the 2012 classification by the Swiss Federal Statistical Office. Categories were relabeled for clarity. Group 0 = no travel; groups 1–10 = deciles among the traveling population.

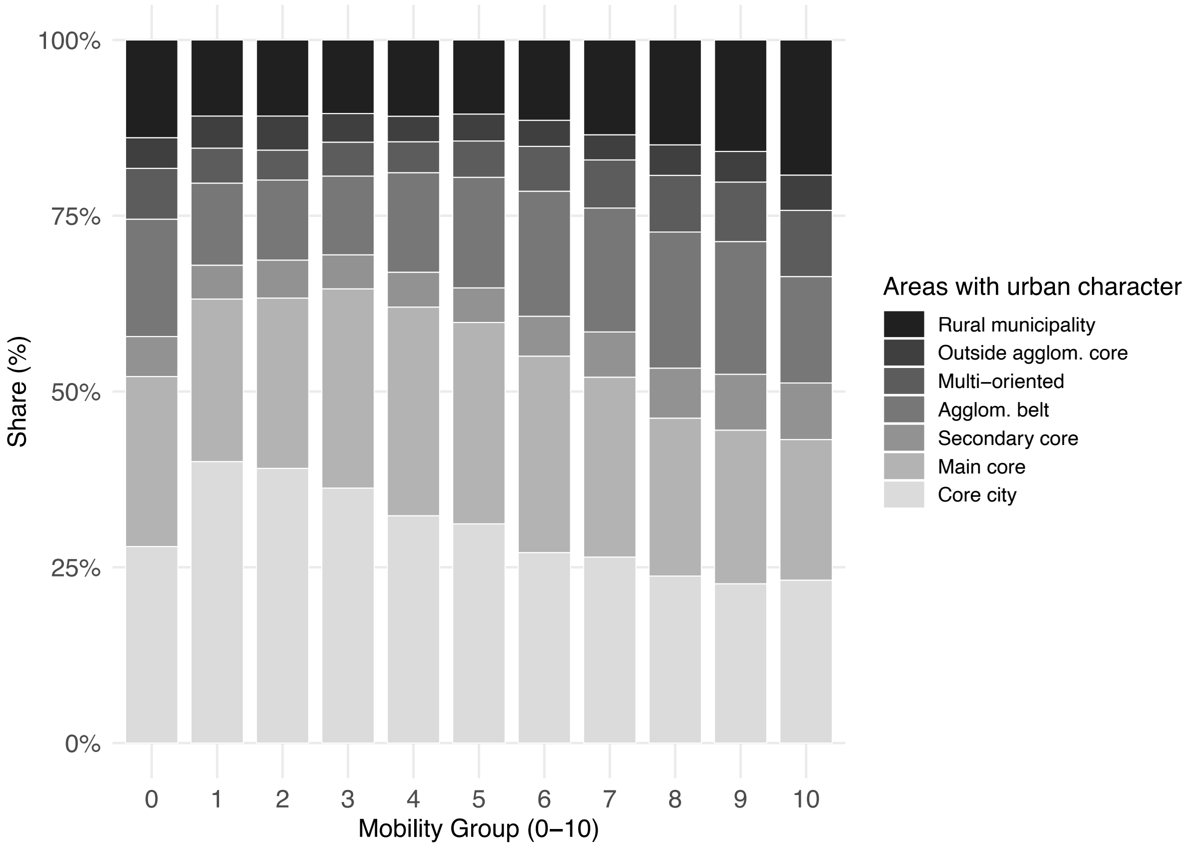

Figure S2

Relative distribution of urban types across mobility groups for the year 2021. Urban types are based on the 2012 classification by the Swiss Federal Statistical Office. Categories were relabeled for clarity. Group 0 = no travel; groups 1–10 = deciles among the traveling population.

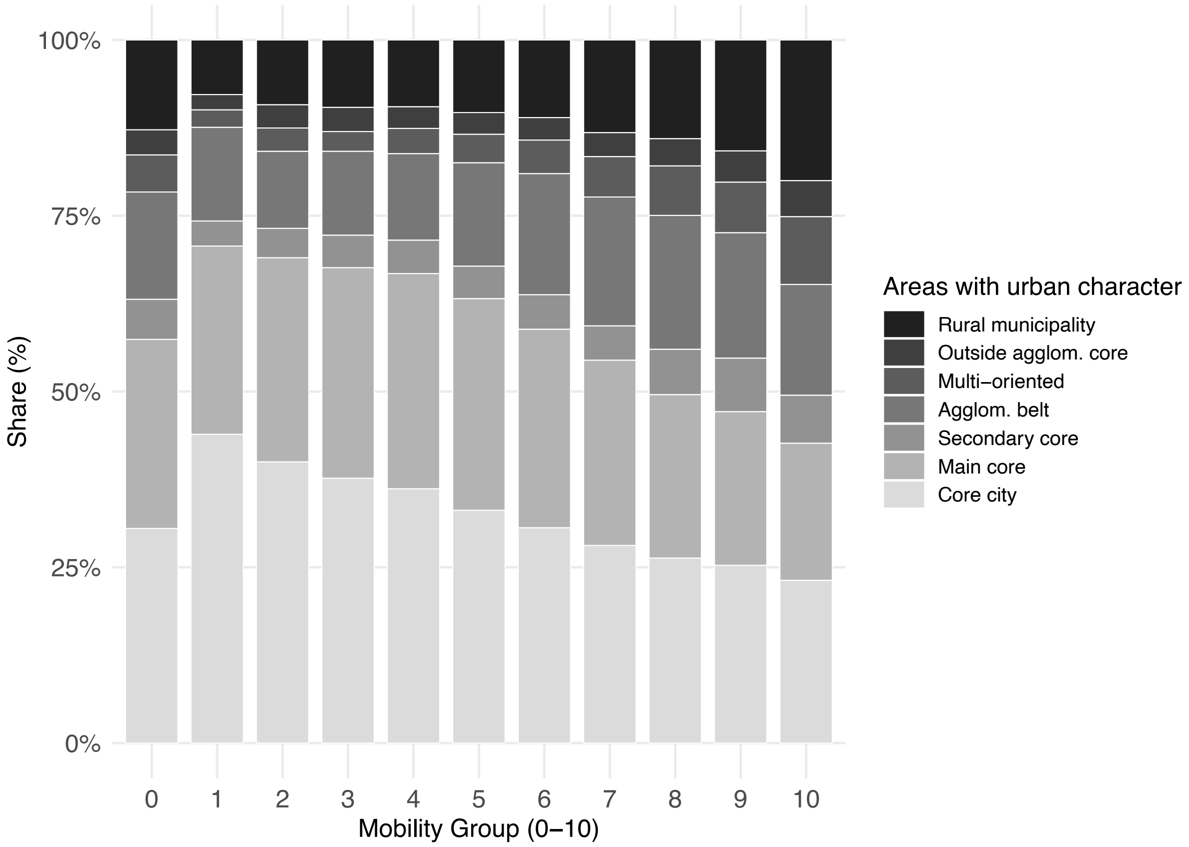

Figure S3

Average daily domestic mobility in km on the municipality level for the year 2015. Total N=56,109. Analysis based on the weighted MTMC data. Minimum sample size n=10 per municipality. White-dotted municipalities were excluded from the illustration due to a small sample size (n<10). No data available for hatched municipalities.

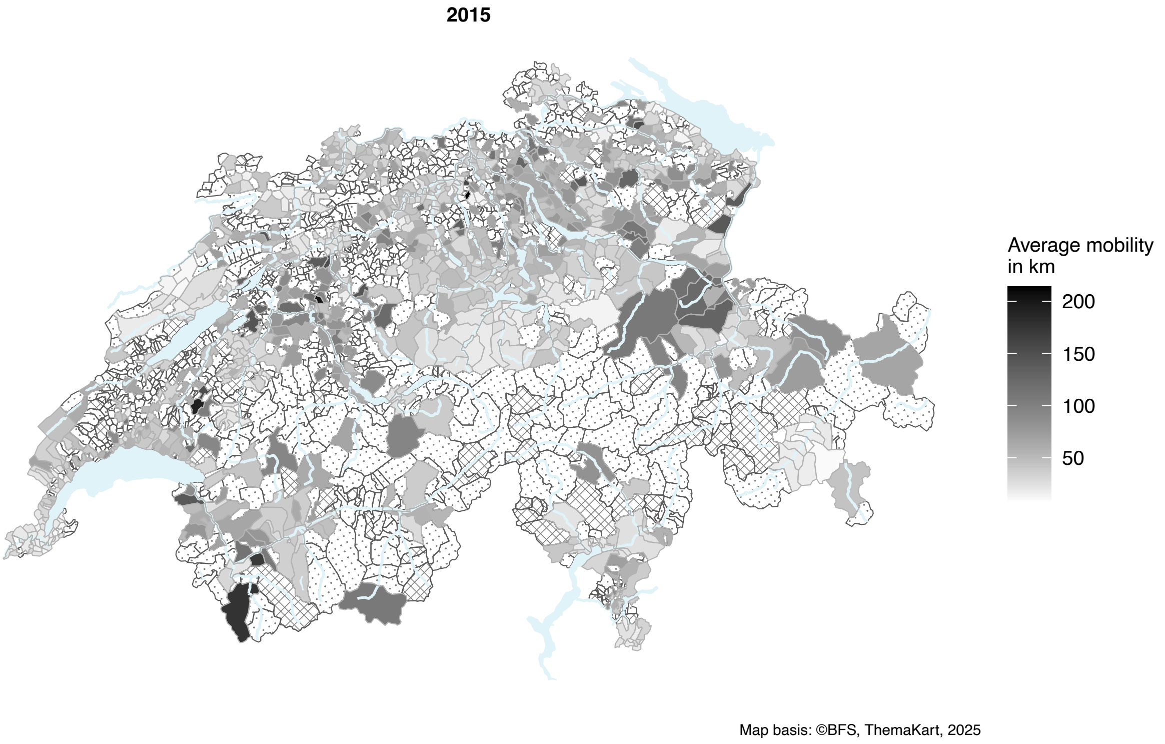

Figure S4

Average daily domestic mobility in km on the municipality level for the year 2015, including metropolitan regions. Total N=56,109. Analysis based on the weighted MTMC data. Minimum sample size n=10 per municipality. White-dotted municipalities were excluded from the illustration due to a small sample size (n<10). No data available for hatched municipalities.

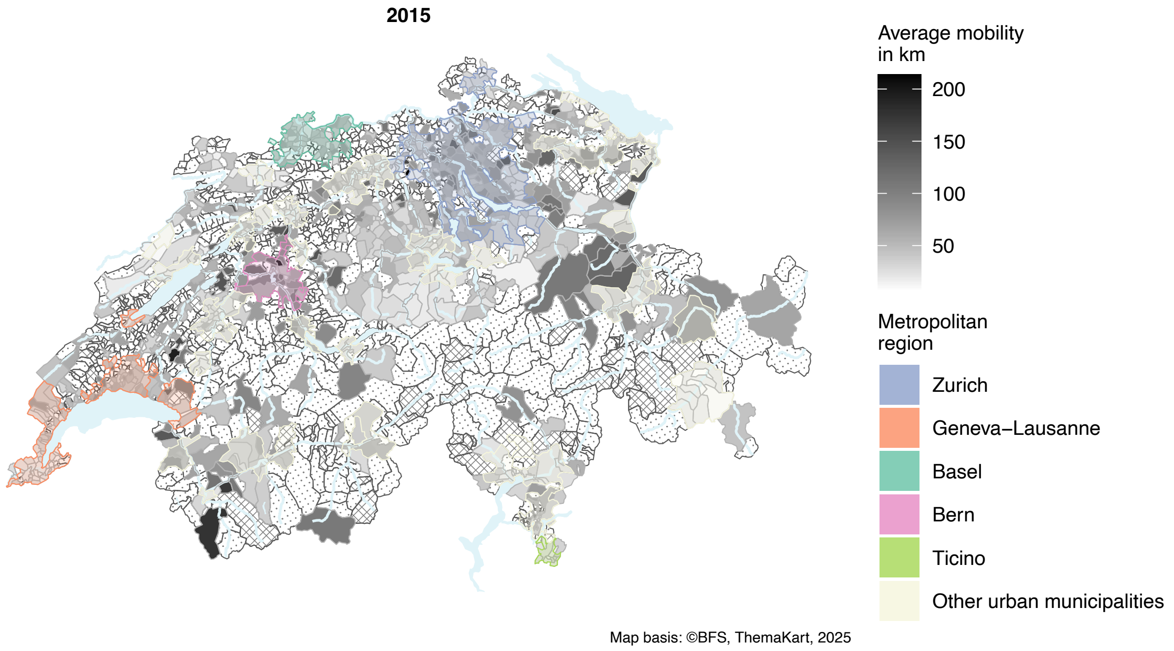

Figure S5

Average daily domestic mobility in km on the municipality level for the year 2021. Total N=54,006. Analysis based on the weighted MTMC data. Minimum sample size n=10 per municipality. White-dotted municipalities were excluded from the illustration due to a small sample size (n<10). No data available for hatched municipalities.

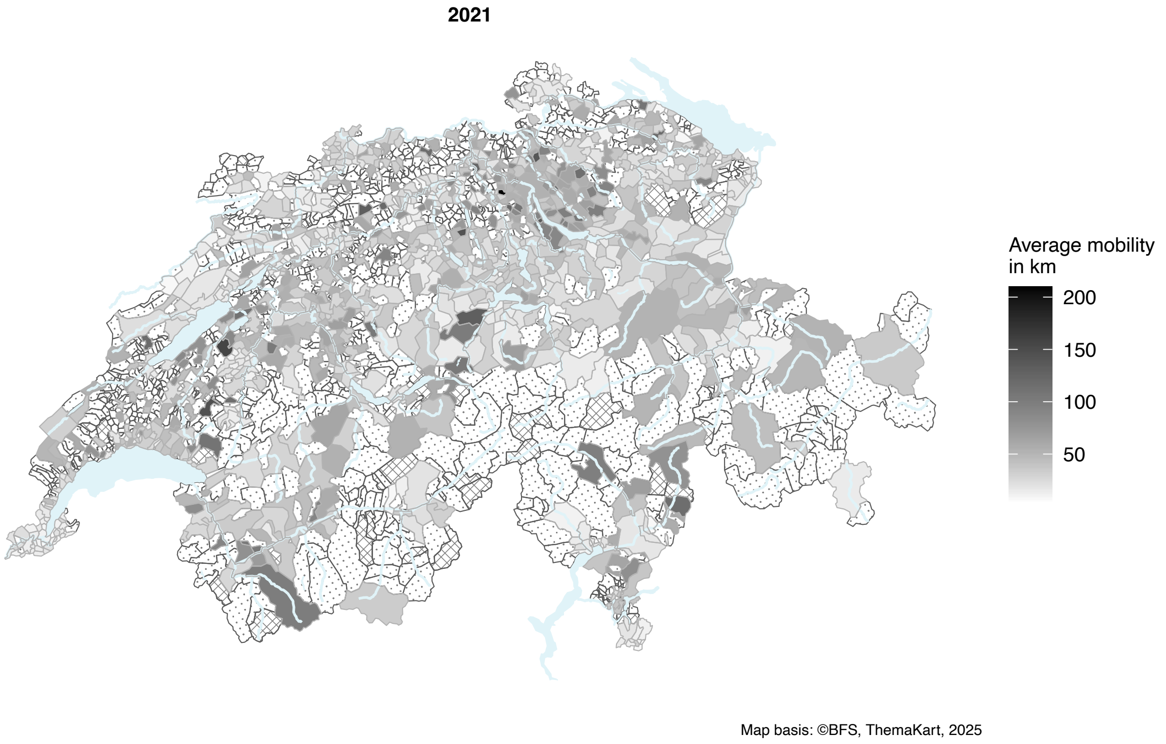

Figure S6

Average daily domestic mobility in km on the municipality level for the year 2021, including metropolitan regions. Total N=54,006. Analysis based on the weighted MTMC data. Minimum sample size n=10 per municipality. White-dotted municipalities were excluded from the illustration due to a small sample size (n<10). No data available for hatched municipalities.

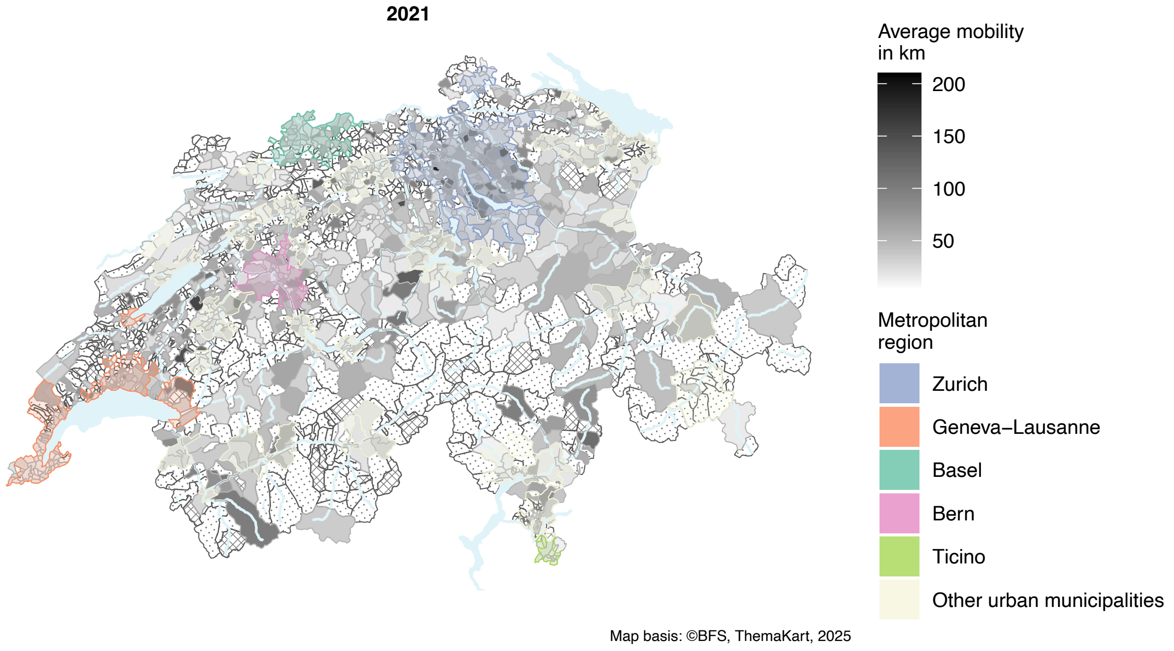

Supplementary Materials D

The Uneven Landscape of Swiss Travel Behavior: Evidence of Mobility Inequality from the National Microcensus

Table S6

Descriptive statistics of age (in years) among the groups for the years 2015 and 2021.

Group	Min	Q1	Q2	Q3	Max	M	SD	n
				**2015**				
0	6	34.00	55	71	103	51.77	23.42	6,649
1	6	15.00	49	68	99	44.42	25.95	5,045
2	6	20.00	48	65	96	44.96	24.04	5,044
3	6	23.75	47	64	96	44.78	22.89	5,044
4	6	26.00	48	63	97	45.48	22.16	5,044
5	6	28.00	47	61	96	44.83	20.67	5,044
6	6	27.00	45	58	95	43.51	19.90	5,044
7	6	28.75	45	58	91	43.84	19.29	5,044
8	6	28.00	44	56	94	42.76	18.33	5,044
9	6	27.00	43	55	95	42.01	17.76	5,044
10	6	28.00	41	54	92	41.81	17.17	5,044
				**2021**				
0	6	35	56	73	98	52.84	23.61	9,581
1	6	27	57	72	98	50.78	25.89	4,544
2	6	23	53	69	98	47.77	24.99	4,544
3	6	28	52	67	96	47.85	23.46	4,544
4	6	29	51	65	95	47.21	22.73	4,544
5	6	29	50	64	93	46.94	21.83	4,544
6	6	31	49	62	91	46.57	20.78	4,544
7	6	32	48	61	91	46.19	19.91	4,544
8	6	31	47	59	93	45.21	18.98	4,543
9	6	29	44	57	94	43.64	18.49	4,543
10	6	27	41	55	94	41.57	17.50	4,543

Note. Group 0 = no travel; groups 1–10 = deciles among the traveling population.

Table S7

Frequencies of gender across the years 2015 and 2021.

Group	2015		2021	
	Male	*Female*	*Male*	*Female*
0	2,876	3,773	4,337	5,244
1	2,173	2,872	1,984	2,560
2	2,214	2,830	2,102	2,442
3	2,329	2,715	2,070	2,474
4	2,395	2,649	2,153	2,391
5	2,423	2,621	2,174	2,370
6	2,544	2,500	2,280	2,264
7	2,599	2,445	2,336	2,208
8	2,656	2,388	2,350	2,193
9	2,768	2,276	2,361	2,182
10	2,966	2,078	2,633	1,910

Note. Group 0 = no travel; groups 1–10 = deciles among the traveling population.

Table S8

Frequencies of education levels among the groups for the years 2015 and 2021.

Group	Below 15 years	Primary education	Secondary education	Tertiary education	NA
			**2015**		
0	631	1,385	3,208	1,349	76
1	1,216	959	2,019	820	31
2	979	815	2,199	1,016	35
3	772	861	2,175	1,203	33
4	604	845	2,310	1,259	26
5	485	818	2,338	1,382	21
6	424	777	2,361	1,461	21
7	353	671	2,460	1,535	25
8	318	621	2,420	1,665	20
9	242	577	2,464	1,746	15
10	182	447	2,436	1,964	15
			**2021**		
0	854	1,781	4,260	2,583	103
1	841	723	1,807	1,141	32
2	860	636	1,761	1,262	25
3	712	643	1,822	1,334	33
4	612	660	1,802	1,431	39
5	520	600	1,883	1,518	23
6	419	609	1,938	1,562	16
7	329	572	2,021	1,603	19
8	275	574	2,016	1,656	22
9	247	538	1,990	1,748	20
10	194	444	1,942	1,942	21

Note. Group 0 = no travel; groups 1–10 = deciles among the traveling population.

Table S9

Frequencies of occupational status among the groups for the years 2015 and 2021.

Group	1	2	3	4	5	6	7	8	9	10	NA
**2015**											
0	465	134	1,880	107	158	248	2,407	184	299	126	641
1	315	74	1,176	81	114	190	1,510	104	190	73	1,218
2	337	70	1,571	78	111	187	1,321	84	238	66	981
3	338	54	1,903	114	107	234	1,175	77	215	53	774
4	365	81	2,072	131	126	237	1,137	63	161	63	608
5	382	71	2,350	145	133	236	959	51	172	57	488
6	385	69	2,575	166	113	239	800	50	157	62	428
7	402	66	2,723	170	96	194	777	57	148	55	356
8	400	74	2,935	179	99	199	606	34	125	71	322
9	430	58	3,084	225	103	185	536	22	105	53	243
10	424	51	3,203	146	109	193	547	19	97	71	184
**2021**											
0	570	147	2,961	192	170	368	3,596	212	317	188	864
1	255	44	1,108	46	77	136	1,759	73	134	67	715
2	254	42	1,380	65	85	139	1,445	78	136	56	613
3	286	55	1,605	65	90	157	1,297	65	139	70	523
4	340	53	1,800	92	84	199	1,135	43	123	62	425
5	370	46	1,934	114	71	219	1,037	49	119	62	330
6	361	52	2,173	112	82	194	929	51	112	53	276
7	382	66	2,443	129	66	174	775	45	79	55	248
8	384	48	2,591	162	71	166	654	47	96	48	196
9	396	41	2,697	166	71	172	583	31	77	61	864
10	385	46	2,917	157	72	148	454	17	84	67	715

Note. 1=Self-employed; 2=Family member working in the family business; 3=Employee; 4=Apprentice; 5=Unemployed; 6=Non-working person in education/training; 7=Non-working person in retirement; 8=Disabled non-working person; 9=Non-working housewife/non-working househusband; 10=Other non-working person (if >= 15 years old). NA refers to individuals who did not respond to the question, were unaware of their status, or were under 15. Group 0 = no travel; groups 1–10 = deciles among the traveling population.

Table S10

Descriptive statistics of household size among the groups for the years 2015 and 2021.

Group	Min	Q1	Q2	Q3	Max	M	SD	n
				**2015**				
0	1	2	2	4	13	2.65	1.35	6,649
1	1	2	3	4	9	2.93	1.41	5,045
2	1	2	3	4	14	2.96	1.42	5,044
3	1	2	3	4	13	2.91	1.41	5,044
4	1	2	3	4	11	2.94	1.39	5,044
5	1	2	3	4	11	2.93	1.38	5,044
6	1	2	3	4	17	2.95	1.38	5,044
7	1	2	3	4	11	2.86	1.34	5,044
8	1	2	3	4	10	2.92	1.37	5,044
9	1	2	3	4	11	2.84	1.36	5,044
10	1	2	2	4	9	2.74	1.33	5,044
				**2021**				
0	1	2	2.0	4	10	2.62	1.36	9,581
1	1	2	2.0	4	12	2.73	1.40	4,544
2	1	2	2.0	4	12	2.84	1.39	4,544
3	1	2	2.0	4	10	2.82	1.37	4,544
4	1	2	2.5	4	9	2.83	1.36	4,544
5	1	2	3.0	4	10	2.85	1.37	4,544
6	1	2	3.0	4	9	2.83	1.34	4,544
7	1	2	3.0	4	10	2.83	1.32	4,544
8	1	2	3.0	4	13	2.85	1.35	4,543
9	1	2	3.0	4	11	2.84	1.35	4,543
10	1	2	2.0	4	10	2.75	1.34	4,543

Note. Calculation of the descriptive statistics based on the unweighted MTMC data. Group 0 = no travel; groups 1–10 = deciles among the traveling population.

Table S11

Frequencies of income groups (in CHF) among the groups for the years 2015 and 2021.

Group	< 2,000	2,000-4,000	4,001-6000	6,001-8,000	8,001-10,000	10,001-12,000	12,001-14,000	14,001-16,000	> 16,000	NA
					**2015**					
0	177	1,089	1,053	784	568	346	174	161	219	2,078
1	124	703	686	548	343	226	100	96	113	2,106
2	98	549	756	641	458	287	131	108	171	1,845
3	86	554	746	661	455	323	174	144	205	1,696
4	69	477	725	669	530	327	197	169	264	1,617
5	69	464	740	708	570	428	204	186	267	1,408
6	61	420	670	727	611	411	250	213	315	1,366
7	59	354	730	768	666	447	250	232	315	1,223
8	66	323	638	765	673	485	304	243	373	1,174
9	46	283	639	785	644	574	331	251	398	1,093
10	41	294	718	758	642	565	327	281	458	960
					**2021**					
0	216	1,357	1,390	1,038	823	522	301	253	466	3,215
1	98	649	603	488	348	253	109	109	151	1,736
2	75	496	609	544	386	238	152	121	174	1,749
3	70	482	589	549	374	305	165	147	228	1,635
4	56	426	593	539	444	320	200	172	287	1,507
5	44	409	540	589	487	345	196	158	286	1,490
6	51	353	593	574	489	389	211	188	325	1,371
7	50	357	592	606	536	399	230	205	351	1,218
8	53	292	556	589	616	438	245	222	355	1,177
9	36	305	565	614	596	420	263	248	385	1,111
10	44	218	551	613	566	517	300	262	449	1,023

Note. Income refers to gross household income in CHF. NA refers to individuals who did not respond to the question, were unaware of their household income, or were under 18. Group 0 = no travel; groups 1–10 = deciles among the traveling population.

Table S12

Descriptive statistics of the number of cars per household among the groups for the years 2015 and 2021.

Group	Min	Q1	Q2	Q3	Max	M	SD	n
				**2015**				
0	0	1	1	2	8	1.25	0.90	6,644
1	0	1	1	2	6	1.19	0.83	5,040
2	0	1	1	2	8	1.29	0.86	5,035
3	0	1	1	2	8	1.29	0.86	5,040
4	0	1	1	2	7	1.36	0.89	5,038
5	0	1	1	2	7	1.41	0.89	5,039
6	0	1	1	2	8	1.48	0.91	5,038
7	0	1	1	2	8	1.50	0.91	5,043
8	0	1	1	2	7	1.55	0.92	5,037
9	0	1	1	2	8	1.53	0.92	5,036
10	0	1	1	2	8	1.49	0.94	5,036
				**2021**				
0	0	1	1	2	7	1.24	0.89	9,568
1	0	1	1	2	7	1.18	0.86	4,541
2	0	1	1	2	7	1.25	0.85	4,535
3	0	1	1	2	6	1.26	0.84	4,538
4	0	1	1	2	7	1.32	0.86	4,541
5	0	1	1	2	6	1.38	0.87	4,539
6	0	1	1	2	7	1.43	0.91	4,537
7	0	1	1	2	7	1.49	0.90	4,535
8	0	1	1	2	7	1.53	0.92	4,538
9	0	1	1	2	7	1.52	0.89	4,532
10	0	1	1	2	7	1.52	0.96	4,532

Note. Group 0 = no travel; groups 1–10 = deciles among the traveling population. Participants with more than eight cars were excluded (99.9th percentile).

Table S13

Descriptive statistics of the number of bicycles per household among the groups for the years 2015 and 2021.

Group	Min	Q1	Q2	Q3	Max	M	SD	NA	n
					**2015**				
0	0	0	0	2	7	0.94	1.34	4,679	1,970
1	0	0	1	2	7	1.33	1.60	3,567	1,478
2	0	0	1	2	7	1.37	1.56	3,549	1,495
3	0	0	1	2	7	1.30	1.51	3,550	1,494
4	0	0	1	2	7	1.37	1.60	3,516	1,528
5	0	0	1	2	7	1.31	1.50	3,505	1,539
6	0	0	1	2	7	1.37	1.57	3,551	1,493
7	0	0	1	2	7	1.32	1.49	3,536	1,508
8	0	0	1	2	7	1.43	1.54	3,500	1,544
9	0	0	1	2	7	1.38	1.50	3,589	1,455
10	0	0	1	2	7	1.38	1.50	3,548	1,496
					**2021**				
0	0	0	0	2	7	1.10	1.50	8,304	1,277
1	0	0	1	2	7	1.34	1.58	3,147	1,397
2	0	0	1	2	7	1.48	1.62	3,209	1,335
3	0	0	1	2	7	1.47	1.63	3,249	1,295
4	0	0	1	2	7	1.57	1.66	3,128	1,416
5	0	0	1	2	7	1.52	1.61	3,222	1,322
6	0	0	1	2	7	1.54	1.59	3,200	1,344
7	0	0	1	2	7	1.43	1.53	3,151	1,393
8	0	0	1	2	7	1.59	1.61	3,199	1,344
9	0	0	1	2	7	1.54	1.60	3,266	1,277
10	0	0	1	2	7	1.45	1.51	3,146	1,397

Note. Group 0 = no travel; groups 1–10 = deciles among the traveling population. Participants with more than seven bicycles were excluded (99.9th percentile).

Table S14

Frequencies of public transport quality classification (ARE) among the groups for the years 2015 and 2021.

Group	1	2	3	4	5
			**2015**		
0	840	1,176	1,525	1,766	1,342
1	914	1,002	1,303	1,254	572
2	795	1,067	1,292	1,275	615
3	859	1,037	1,262	1,206	680
4	832	1,028	1,203	1,244	737
5	799	973	1,205	1,301	766
6	632	976	1,145	1,400	891
7	642	834	1,222	1,422	924
8	510	812	1,192	1,562	968
9	487	861	1,171	1,519	1,006
10	650	856	1,103	1,450	985
			**2021**		
0	1,337	1,787	2,391	2,511	1,555
1	846	963	1,256	1,048	431
2	825	891	1,259	1,071	498
3	801	917	1,233	1,085	508
4	824	903	1,155	1,080	582
5	778	868	1,094	1,166	638
6	710	855	1,111	1,170	698
7	597	808	1,108	1,240	791
8	503	750	1,123	1,330	837
9	468	756	1,163	1,338	818
10	550	813	1,013	1,318	849

Note. 1=Class A: Very good accessibility; 2=Class B: Good access; 3=Class C: Average access; 4=Class D: Poor access; 5=No quality class: Marginal or no public transport access. Group 0 = no travel; groups 1–10 = deciles among the traveling population.

Table S15

Frequencies of classification of "areas with urban character 2012" (FSO) among the groups for the years 2015 and 2021.

Group	0	1	2	3	4	5	6
				**2015**			
0	925	1,858	1,608	378	1,109	481	290
1	546	2,019	1,167	242	589	251	231
2	546	1,970	1,222	272	575	214	245
3	528	1,829	1,429	244	565	243	206
4	548	1,630	1,497	250	714	222	183
5	532	1,572	1,445	248	793	261	193
6	577	1,366	1,409	286	897	322	187
7	681	1,334	1,290	325	889	345	180
8	753	1,198	1,132	359	977	406	219
9	800	1,142	1,102	401	952	427	220
10	970	1,168	1,009	406	763	475	253
				**2021**			
0	1,225	2,924	2,576	546	1,463	505	342
1	353	1,995	1,217	162	605	113	99
2	419	1,817	1,319	190	498	151	150
3	436	1,711	1,361	210	542	128	156
4	432	1,643	1,391	216	559	163	140
5	469	1,504	1,368	210	668	184	141
6	502	1,391	1,283	223	783	217	145
7	599	1,277	1,197	222	833	261	155
8	638	1,195	1,056	293	865	320	176
9	717	1,148	993	346	810	327	202
10	909	1,051	886	310	716	438	233

Note. 0= Rural municipality without urban character; 1=Core municipality of an agglomeration (core city); 2=Core municipality of an agglomeration (main core); 3=Core municipality of an agglomeration (secondary core); 4=Municipality in the agglomeration belt; 5=Multi-oriented municipality; 6=Core municipality outside agglomerations. Group 0 = no travel; groups 1–10 = deciles among the traveling population.

Table S16

Descriptive statistics of the daily number of commuting-related trips among the groups for the years 2015 and 2021.

Group	Min	Q1	Q2	Q3	Max	M	SD	n
				**2015**				
0	0	0	0	0	0	0	0	6,649
1	0	0	0	0	11.76	0.18	0.69	5,045
2	0	0	0	0	12.01	0.31	0.89	5,044
3	0	0	0	0	12.17	0.43	1.01	5,044
4	0	0	0	0.66	12.90	0.54	1.15	5,044
5	0	0	0	1.15	16.68	0.69	1.32	5,044
6	0	0	0	1.39	15.74	0.83	1.46	5,044
7	0	0	0	1.60	16.72	0.97	1.65	5,044
8	0	0	0	2.00	19.64	1.22	1.91	5,044
9	0	0	0	2.41	20.71	1.40	2.16	5,044
10	0	0	0	2.63	20.01	1.51	2.55	5,044
				**2021**				
0	0	0	0	0	0	0	0	9,581
1	0	0	0	0	24.08	0.14	0.69	4,544
2	0	0	0	0	8.50	0.18	0.61	4,544
3	0	0	0	0	17.05	0.32	0.96	4,544
4	0	0	0	0	13.96	0.40	1.00	4,544
5	0	0	0	0.64	15.08	0.54	1.20	4,544
6	0	0	0	1.00	16.90	0.69	1.40	4,544
7	0	0	0	1.42	19.93	0.88	1.58	4,544
8	0	0	0	1.70	16.36	1.04	1.72	4,543
9	0	0	0	2.14	20.60	1.28	2.05	4,543
10	0	0	0	3.05	21.84	1.75	2.77	4,543

Note. Calculation of the descriptive statistics based on the unweighted MTMC data. Group 0 = no travel; groups 1–10 = deciles among the traveling population.

Table S17

Descriptive statistics of the daily number of education-related trips among the groups for the years 2015 and 2021.

Group	Min	Q1	Q2	Q3	Max	M	SD	n
				**2015**				
0	0	0	0	0	0	0	0	6,649
1	0	0	0	0	8.50	0.28	0.74	5,045
2	0	0	0	0	10.18	0.35	0.98	5,044
3	0	0	0	0	16.67	0.35	1.08	5,044
4	0	0	0	0	11.80	0.28	0.94	5,044
5	0	0	0	0	20.65	0.28	1.00	5,044
6	0	0	0	0	20.26	0.29	1.11	5,044
7	0	0	0	0	23.49	0.27	1.11	5,044
8	0	0	0	0	14.57	0.24	0.96	5,044
9	0	0	0	0	23.29	0.24	1.01	5,044
10	0	0	0	0	19.46	0.24	1.08	5,044
				**2021**				
0	0	0	0	0	0	0	0	9,581
1	0	0	0	0	7.19	0.17	0.57	4,544
2	0	0	0	0	10.32	0.29	0.87	4,544
3	0	0	0	0	12.09	0.33	1.00	4,544
4	0	0	0	0	11.74	0.33	1.07	4,544
5	0	0	0	0	22.36	0.31	1.17	4,544
6	0	0	0	0	13.13	0.27	1.04	4,544
7	0	0	0	0	12.17	0.23	0.98	4,544
8	0	0	0	0	22.36	0.21	1.00	4,543
9	0	0	0	0	16.16	0.20	0.93	4,543
10	0	0	0	0	16.25	0.23	1.08	4,543

Note. Calculation of the descriptive statistics based on the unweighted MTMC data. Group 0 = no travel; groups 1–10 = deciles among the traveling population.

Table S18

Descriptive statistics of the daily number of shopping-related trips among the groups for the years 2015 and 2021.

Group	Min	Q1	Q2	Q3	Max	M	SD	n
				**2015**				
0	0	0	0	0	0	0	0	6,649
1	0	0	0	0.82	8.53	0.50	0.88	5,045
2	0	0	0	0.99	9.79	0.63	1.05	5,044
3	0	0	0	1.18	15.40	0.73	1.20	5,044
4	0	0	0	1.24	13.88	0.80	1.30	5,044
5	0	0	0	1.35	13.32	0.86	1.43	5,044
6	0	0	0	1.41	15.70	0.90	1.47	5,044
7	0	0	0	1.47	25.19	0.99	1.65	5,044
8	0	0	0	1.45	19.48	0.97	1.67	5,044
9	0	0	0	1.51	23.36	1.03	1.88	5,044
10	0	0	0	1.51	26.12	1.08	2.16	5,044
				**2021**				
0	0	0	0	0	0	0	0	9,581
1	0	0	0	0.71	7.77	0.43	0.76	4,544
2	0	0	0	0.83	14.76	0.54	1.02	4,544
3	0	0	0	1.03	21.33	0.65	1.17	4,544
4	0	0	0	1.22	10.82	0.76	1.21	4,544
5	0	0	0	1.31	17.16	0.84	1.36	4,544
6	0	0	0	1.40	10.68	0.90	1.44	4,544
7	0	0	0	1.49	17.65	0.96	1.62	4,544
8	0	0	0	1.54	33.27	1.05	1.89	4,543
9	0	0	0	1.60	19.10	1.11	1.95	4,543
10	0	0	0	1.60	32.40	1.20	2.45	4,543

Note. Calculation of the descriptive statistics based on the unweighted MTMC data. Group 0 = no travel; groups 1–10 = deciles among the traveling population.

Table S19

Descriptive statistics of the daily number of business-related trips among the groups for the years 2015 and 2021.

Group	Min	Q1	Q2	Q3	Max	M	SD	n
				**2015**				
0	0	0	0	0	0	0	0	6,649
1	0	0	0	0	7.09	0.01	0.18	5,045
2	0	0	0	0	5.43	0.02	0.18	5,044
3	0	0	0	0	9.04	0.04	0.28	5,044
4	0	0	0	0	10.75	0.06	0.40	5,044
5	0	0	0	0	12.56	0.08	0.48	5,044
6	0	0	0	0	8.11	0.09	0.51	5,044
7	0	0	0	0	10.71	0.14	0.64	5,044
8	0	0	0	0	12.67	0.19	0.80	5,044
9	0	0	0	0	22.99	0.25	0.99	5,044
10	0	0	0	0	27.99	0.47	1.61	5,044
				**2021**				
0	0	0	0	0	0	0	0	9,581
1	0	0	0	0	15.41	0.01	0.24	4,544
2	0	0	0	0	4.99	0.01	0.14	4,544
3	0	0	0	0	4.63	0.01	0.16	4,544
4	0	0	0	0	6.53	0.01	0.17	4,544
5	0	0	0	0	6.28	0.03	0.25	4,544
6	0	0	0	0	9.02	0.03	0.30	4,544
7	0	0	0	0	9.82	0.05	0.42	4,544
8	0	0	0	0	16.92	0.07	0.50	4,543
9	0	0	0	0	13.07	0.09	0.57	4,543
10	0	0	0	0	11.05	0.18	0.91	4,543

Note. Calculation of the descriptive statistics based on the unweighted MTMC data. Group 0 = no travel; groups 1–10 = deciles among the traveling population.

Table S20

Descriptive statistics of the daily number of leisure-related trips among the groups for the years 2015 and 2021.

Group	Min	Q1	Q2	Q3	Max	M	SD	n
				**2015**				
0	0	0	0	0	0	0	0	6,649
1	0	0	0.38	0.96	8.37	0.63	0.89	5,045
2	0	0	0.59	1.25	11.39	0.86	1.13	5,044
3	0	0	0.76	1.51	11.22	1.02	1.21	5,044
4	0	0	0.86	1.74	14.88	1.22	1.50	5,044
5	0	0	0.99	1.96	14.96	1.34	1.59	5,044
6	0	0	1.06	2.15	18.42	1.45	1.74	5,044
7	0	0	1.12	2.43	22.60	1.63	1.95	5,044
8	0	0	1.18	2.59	19.58	1.75	2.17	5,044
9	0	0	1.39	2.90	20.38	2.03	2.46	5,044
10	0	0	1.95	3.82	35.56	2.73	3.17	5,044
				**2021**				
0	0	0	0	0	0	0	0	9,581
1	0	0	0.27	0.71	9.18	0.48	0.71	4,544
2	0	0	0.52	1.02	8.30	0.70	0.89	4,544
3	0	0	0.59	1.30	10.54	0.85	1.08	4,544
4	0	0	0.62	1.45	11.06	0.95	1.19	4,544
5	0	0	0.69	1.64	13.88	1.08	1.42	4,544
6	0	0	0.77	1.83	16.45	1.22	1.61	4,544
7	0	0	0.81	2.04	22.89	1.35	1.85	4,544
8	0	0	0.92	2.14	20.95	1.48	2.01	4,543
9	0	0	1.07	2.43	23.88	1.67	2.20	4,543
10	0	0	1.55	3.54	33.63	2.41	3.10	4,543

Note. Calculation of the descriptive statistics based on the unweighted MTMC data. Group 0 = no travel; groups 1–10 = deciles among the traveling population.

Table S21

Descriptive statistics of the daily number of accompanying-related trips among the groups for the years 2015 and 2021.

Group	Min	Q1	Q2	Q3	Max	M	SD	n
				**2015**				
0	0	0	0	0	0	0	0	6,649
1	0	0	0	0	5.43	0.04	0.27	5,045
2	0	0	0	0	7.13	0.09	0.48	5,044
3	0	0	0	0	10.28	0.12	0.55	5,044
4	0	0	0	0	16.20	0.19	0.75	5,044
5	0	0	0	0	16.04	0.22	0.84	5,044
6	0	0	0	0	11.38	0.24	0.79	5,044
7	0	0	0	0	15.69	0.29	1.00	5,044
8	0	0	0	0	16.08	0.34	1.09	5,044
9	0	0	0	0	23.59	0.38	1.26	5,044
10	0	0	0	0	26.55	0.42	1.43	5,044
				**2021**				
0	0	0	0	0	0	0	0	9,581
1	0	0	0	0	5.20	0.03	0.24	4,544
2	0	0	0	0	5.92	0.06	0.36	4,544
3	0	0	0	0	22.36	0.11	0.63	4,544
4	0	0	0	0	9.36	0.13	0.58	4,544
5	0	0	0	0	13.73	0.16	0.70	4,544
6	0	0	0	0	16.16	0.20	0.81	4,544
7	0	0	0	0	19.88	0.28	1.03	4,544
8	0	0	0	0	29.78	0.34	1.21	4,543
9	0	0	0	0	20.99	0.34	1.21	4,543
10	0	0	0	0	29.71	0.46	1.73	4,543

Note. Calculation of the descriptive statistics based on the unweighted MTMC data. Group 0 = no travel; groups 1–10 = deciles among the traveling population.

Table S22

Frequencies of travel day among the groups for the years 2015 and 2021.

Group	1	2	3	4	5	6	7
				**2015**			
0	950	732	707	651	560	888	2,161
1	955	912	611	593	375	552	1,047
2	895	833	697	642	493	499	985
3	913	856	753	685	519	520	798
4	919	813	751	647	581	572	761
5	856	826	708	751	631	573	699
6	848	818	793	670	602	626	687
7	781	806	793	724	649	618	673
8	789	813	828	712	650	622	630
9	696	730	796	753	724	690	655
10	597	670	690	679	872	827	709
				**2021**			
0	1,425	1,234	1,208	1,069	848	1,321	2,476
1	915	793	650	510	350	485	841
2	815	759	663	543	436	513	815
3	868	810	678	608	416	470	694
4	796	832	671	592	490	497	666
5	771	767	750	597	521	535	603
6	743	762	716	627	524	555	617
7	766	746	707	618	574	561	572
8	739	723	732	640	558	559	592
9	682	708	670	634	611	636	602
10	607	599	614	657	715	700	651

Note. 1=Monday; 2=Tuesday; 3=Wednesday; 4=Thursday; 5=Friday; 6=Saturday; 7=Sunday. Group 0 = no travel; groups 1–10 = deciles among the traveling population.

Table S23
Frequencies of travel card ownership for public transport among the groups for the years 2015 and 2021.

Group	1	2	3	4	5	6	7	8	NA
					**2015**				
0	450	1848	497	95	0	226	55	3476	1848
1	294	1340	301	72	0	474	31	2530	1340
2	282	1502	361	62	1	437	55	2339	1502
3	327	1603	422	69	1	348	37	2234	1603
4	406	1615	483	80	2	238	59	2159	1615
5	366	1673	545	71	0	214	55	2118	1673
6	378	1675	513	85	1	196	33	2160	1675
7	400	1736	436	72	0	162	41	2196	1736
8	482	1725	394	95	0	159	26	2156	1725
9	682	1778	370	68	2	121	18	1998	1778
10	947	1791	252	65	0	99	18	1868	1791
					**2021**				
0	654	2616	718	132	1	223	106	5123	654
1	299	1252	276	48	1	246	36	2385	299
2	331	1328	306	55	2	273	32	2215	331
3	272	1380	349	57	1	214	52	2214	272
4	289	1371	444	72	0	208	57	2100	289
5	285	1443	454	65	2	151	53	2088	285
6	299	1463	405	80	2	119	52	2121	299
7	287	1514	372	73	1	89	58	2144	287
8	400	1540	361	79	0	73	50	2038	400
9	482	1572	311	75	0	67	46	1981	482
10	623	1556	256	47	2	52	32	1968	623

Note. 1= GA Travelcard; 2= Half-Fare Travelcard/Half-Fare Youth Travelcard; 3= Regional Network Pass; 4= Point-to-point Travelcard; 5=seven25 Travelcard/Gleis 7 Card; 6= Junior Travelcard/Children's Co-Travelcard; 7= Other Season Ticket (e.g., Modular Travelcard). Group 0 = no travel; groups 1–10 = deciles among the traveling population.

Supplementary Materials E

The Uneven Landscape of Swiss Travel Behavior: Evidence of Mobility Inequality from the National Microcensus

Table S24

Descriptive statistics of travel distances (in km) among the groups for air travel *for the years 2015 and 2021.*

Group	Min	Q1	Q2	Q3	Max	M	SD	n
				**2015**				
0	164.34	1,239.89	2,541.10	6,005.11	64,246.34	5,832.47	9,125.85	351
1	114.95	1,134.16	2,020.45	4,137.95	66,383.26	4,423.95	7,369.01	243
2	109.28	1,092.20	2,279.67	6,085.31	50,328.71	5,451.57	8,043.85	303
3	265.80	1,045.05	2,219.94	5,577.23	80,093.94	5,369.02	9,257.49	305
4	129.97	1,288.63	2,437.01	7,685.32	68,160.62	6,057.55	8,499.77	358
5	217.74	1,281.56	2,548.37	6,784.82	117,213.47	6,760.92	12,003.63	386
6	257.98	1,235.94	2,728.82	8,065.12	58,756.40	7,231.09	10,350.67	369
7	244.69	1,591.37	3,106.64	7,433.42	97,400.24	7,174.41	11,790.02	358
8	159.74	1,576.12	3,276.70	8,721.23	162,729.62	8,203.01	14,053.48	454
9	395.05	2,123.43	3,959.15	11,685.12	93,316.83	9,280.42	12,159.75	471
10	409.86	2,375.03	5,478.64	15,141.03	136,714.48	11,845.28	16,422.22	476
				**2021**				
0	198.83	1,447.81	2,950.31	5,757.38	39,857.58	5,085.79	6,314.07	163
1	225.59	939.60	1,540.37	3,081.97	26,130.29	2,704.46	3,640.67	87
2	159.02	1,491.36	2,359.16	4,901.37	19,273.73	3,605.56	3,443.16	80
3	253.17	1,205.90	2,149.25	4,270.41	32,131.24	3,746.20	5,288.14	89
4	194.27	1,403.64	2,280.48	3,588.42	48,788.24	4,594.98	8,702.35	78
5	281.98	1,398.65	2,635.06	4,830.17	24,850.49	4,453.08	5,214.42	128
6	230.96	1,953.86	3,291.08	5,429.86	104,890.38	6,221.08	11,278.82	107
7	334.76	1,509.92	2,521.40	4,490.39	47,226.33	4,218.54	5,629.62	125
8	145.28	1,768.95	3,241.47	6,209.70	74,460.15	6,595.97	10,304.50	117
9	47.53	2,081.89	4,017.80	7,176.05	40,306.39	6,068.60	6,265.06	125
10	522.35	2,600.21	4,854.35	7,988.39	44,652.38	7,596.43	8,583.32	140

Note. Values report air-travel distance only (km) and represent the sum of distances for trips from the MTMC overnight-travel module (German: "Reisen mit Übernachtungen"; English: "Trips with overnight stays") in which the airplane was selected as the mode of transport. The overnight-travel module was administered to a subsample (approximately one third of MTMC participants). The 11 groups (0–10) correspond to those used in the main analyses based on daily domestic travel distance (group 0 = no domestic travel; groups 1–10 = deciles among travelers; weighted MTMC data). In the MTMC survey, participants had to select a mode of transport when reporting at least one trip with overnight stays. Since we included only participants with at least one flight in the additional analysis of air travel, all values are greater than zero.

Figure S7

Lorenz curves of international air travel distances for the years 2015 and 2021. The dotted vertical line marks the 90th percentile of the population. Gini values for the year 2015 = 0.62 and for the year 2021 = 0.55, respectively.

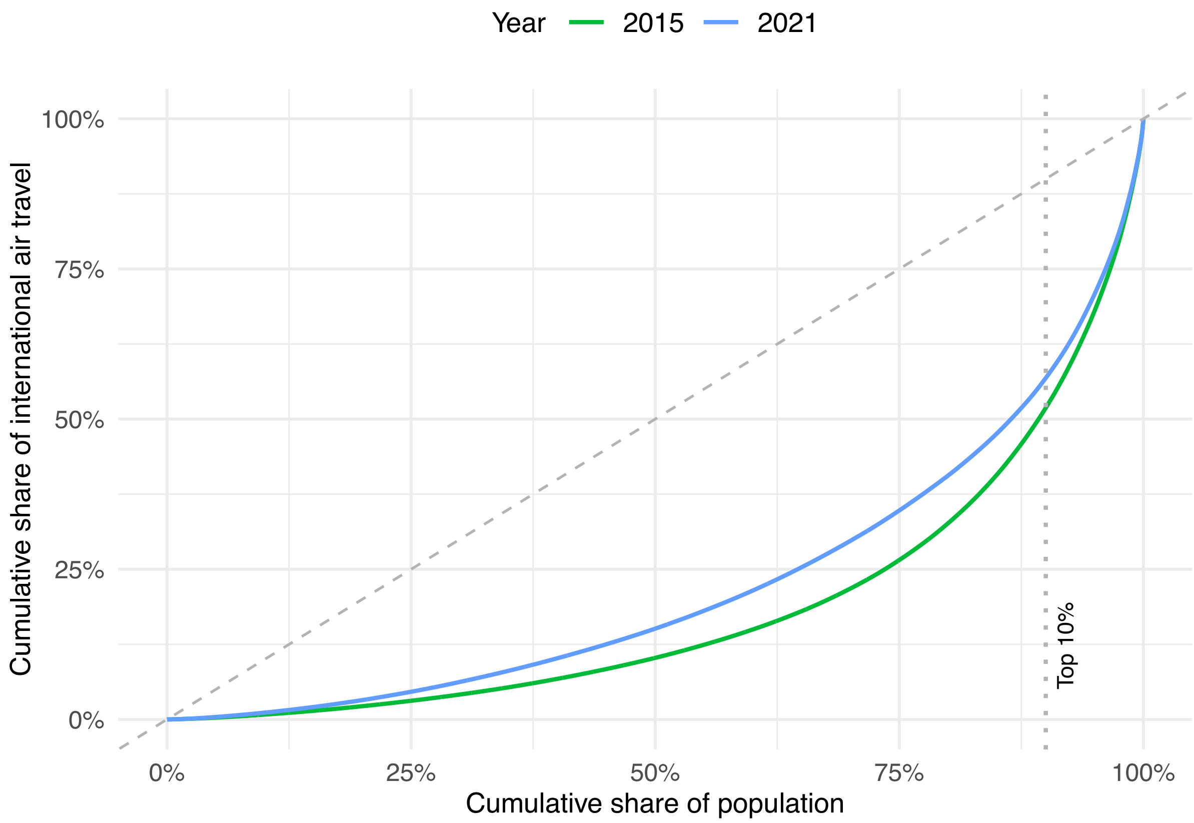

Supplement: Supplementary file 1 — Supplementary Materials [file 44333_2026_85_MOESM1_ESM.pdf]
